
JOURNAL INFORMATION
==============================
NLM Title Abbreviation: Orthop Surg
Journal ID: Orthop Surg
NLM Journal ID: 101501666
Journal ID: OS

Orthopaedic Surgery

ISSN: 1757-7853
EISSN: 1757-7861
Publisher: Wiley

ARTICLE INFORMATION
==============================
PMCID: PMC11474839
DOI: 10.1111/os.13344
Article ID: OS13344
Article version: 1
Subjects: Issue Information, Issue Information

Issue Information

Electronic publication date: 2023 Oct 4
Publication date: 2023 Oct
Volume: 15
Issue: 10
First page: 2483
Last page: 2484
Copyright: © 2023 The Authors. Orthopaedic Surgery published by Tianjin Hospital and John Wiley & Sons Australia, Ltd.
License: This is an open access article under the terms of the http://creativecommons.org/licenses/by/4.0/ License, which permits use, distribution and reproduction in any medium, provided the original work is properly cited.
License URL: https://creativecommons.org/licenses/by/4.0/

Figure count: 0
Table count: 0
Page count: 5

source-schema-version-number: 2.0
cover-date: October 2023
details-of-publishers-convertor: Converter:WILEY_ML3GV2_TO_JATSPMC version:6.4.9 mode:remove_FC converted:15.10.2024

==============================

Volume 15 • Issue 10 • October 2023 • ISSN 1757-7861

### Orthopaedic Surgery

Open Access

REVIEW ARTICLES
2485
THE MOREL-LAVALLÉE LESION: REVIEW AND UPDATE ON DIAGNOSIS AND MANAGEMENT
2492
THERAPEUTIC EFFICACY AND SAFETY OF PERCUTANEOUS CURVED VERTEBROPLASTY IN OSTEOPOROTIC VERTEBRAL COMPRESSION FRACTURES: A SYSTEMATIC REVIEW AND META-ANALYSIS
2505
BIBLIOMETRIC INSIGHTS IN ADVANCES OF CHORDOMA: GLOBAL TRENDS AND RESEARCH DEVELOPMENT IN THE LAST DECADE

CLINICAL ARTICLES
2515
PREVENTION OF BONE CEMENT DISPLACEMENT IN KÜMMELL DISEASE WITHOUT NEUROLOGICAL DEFICITS THROUGH TREATMENT WITH A NOVEL HOLLOW PEDICLE SCREW COMBINED WITH KYPHOPLASTY
2523
DESIGN AND APPLICATION OF AN ACETABULAR INTEGRATIVE ANATOMIC PLATE: A RETROSPECTIVE STUDY OF 178 CASES WITH COMPLEX ACETABULAR FRACTURES
2532
A CLINICAL COMPARATIVE STUDY ON PERCUTANEOUS AND OPEN APPROACHES FOR SCREW INTERNAL FIXATION IN TREATING THORACOLUMBAR FRACTURES
2540
LIMB RECONSTRUCTION SYSTEM ASSISTED REDUCTION AND INTERNAL FIXATION FOR INTRA-ARTICULAR CALCANEAL FRACTURES: A NEW APPLICATION
2549
PREOPERATIVE SKULL TRACTION, ANTERIOR DEBRIDEMENT, BONE GRAFTING, AND INTERNAL FIXATION FOR CERVICAL TUBERCULOSIS WITH SEVERE KYPHOSIS
2557
IN DIFFERENT GENDER GROUPS, WHAT IS THE IMPACT OF THE FIBULAR NOTCH ON THE SEVERITY OF HIGH ANKLE SPRAIN: A RETROSPECTIVE STUDY OF 360 CASES
2566
FEASIBILITY OF ANTERIOR FIXATION WITH SINGLE SCREW FOR ODONTOID FRACTURES IN PEDIATRICS: A COMPUTED TOMOGRAPHIC STUDY
2574
RISK FACTORS OF NONFUSION AFTER ANTERIOR CERVICAL DECOMPRESSION AND FUSION IN THE EARLY POSTOPERATIVE PERIOD: A RETROSPECTIVE STUDY
2582
CLINICAL OUTCOMES OF ISOBAR TTL SYSTEM WITH ISTHMIC BONE GRAFTING AND PEDICLE SCREW-VERTEBRAL PLATE HOOK WITH DIRECT REPAIR OF DEFECT FOR LUMBAR SPONDYLOLYSIS: A MATCHED-PAIR CASE CONTROL STUDY
2591
SIMPLE MINIMALLY INVASIVE METHOD TO REDUCE VALGUS-IMPACTED AND TILTED FEMORAL NECK FRACTURES WITHOUT SOFT TISSUE OR CARTILAGE INJURY: RADIOLOGICAL AND CLINICAL RESULTS
2602
SURGICAL STRATEGY AND OUTCOMES OF FULL ENDOSCOPIC LUMBAR DISCECTOMY FOR RECURRENT LUMBAR DISK HERNIATION FOLLOWING A PREVIOUS FULL ENDOSCOPIC LUMBAR DISCECTOMY
2612
VASTUS LATERALIS AND VASTUS INTERMEDIUS MYOCUTANEOUS FLAP RECONSTRUCTION FOR COMPLICATED TROCHANTERIC AND ISCHIAL PRESSURE SORES WITH EXTENDED GIRDLESTONE RESECTION: A CASE SERIES
2621
COMPARISON OF MODIFIED K-WIRE FIXATION WITH OPEN REDUCTION AND INTERNAL FIXATION (ORIF) FOR UNSTABLE COLLES FRACTURE IN ELDERLY PATIENTS
2627
CORRELATION BETWEEN THE COAPTATION AND REGENERATION OF TENDON STUMPS IN ENDOSCOPIC ASSISTED ACHILLES TENDON RUPTURE REPAIR
2638
SELECTING THE VERTEBRA ABOVE SAGITTAL STABLE VERTEBRA AS THE DISTAL FUSION LEVEL IN SCHEUERMANN'S KYPHOSIS: A PROSPECTIVE STUDY WITH A MINIMUM OF 2-YEAR FOLLOW-UP
2647
COMPARISON OF ANTERIOR CERVICAL DISCECTOMY FUSION COMBINED WITH LATERAL MASS SCREW AND WITH CERVICAL PEDICLE SCREW FIXATION SURGERY UNDER O-ARM NAVIGATION FOR SINGLE-STAGE MANAGEMENT OF SEVERE LOWER CERVICAL FRACTURE DISLOCATION
2656
MINI-OPEN PEDICLE SUBTRACTION OSTEOTOMY VERSUS STANDARD POSTERIOR APPROACH FOR ANKYLOSING SPONDYLITIS-RELATED SPINAL KYPHOSIS: A COMPARATIVE STUDY
2665
LATERAL CENTER-EDGE ANGLE OF 18° (BONE-EDGE): THRESHOLD FOR HIP ARTHROSCOPY TREATMENT IN PATIENTS WITH BORDERLINE DEVELOPMENTAL DYSPLASIA OF THE HIP?

RESEARCH ARTICLES
2674
ANALYSIS OF THE ABILITY OF A DISTAL TIBIAL ANATOMICAL LOCKING PLATE TO CAPTURE THE DISTAL TIBIAL FRAGMENTS IN PATIENTS WITH PILON FRACTURES
2683
THE SPECIFIC ANATOMICAL MORPHOLOGY OF LATERAL ANKLE LIGAMENT: QUALITATIVE AND QUANTITATIVE CADAVERIC BASED STUDY
2689
THE EFFECT OF THE HIP FLEXION ANGLE IN OSTEONECROSIS OF THE FEMORAL HEAD BASED ON CHINA-JAPAN FRIENDSHIP HOSPITAL CLASSIFICATION - A FINITE ELEMENT STUDY
2701
BIOMECHANICAL COMPARISON OF DIFFERENT SURGICAL APPROACHES FOR THE TREATMENT OF ADJACENT SEGMENT DISEASES AFTER PRIMARY TRANSFORAMINAL LUMBAR INTERBODY FUSION: A FINITE ELEMENT ANALYSIS

OPERATIVE TECHNIQUES
2709
CONGENITAL CERVICAL SCOLIOSIS TREATED WITH CONCAVE SIDE DISTRACTION WITH THREE-DIMENSIONAL PRINTED TITANIUM CAGE
2716
RECONSTRUCTION OF COMPOSITE SOFT TISSUE DEFECT IN THE DISTAL FINGER USING PARTIAL TOENAIL FLAP TRANSFER

CASE REPORTS
2724
USING 3D PRINTING TECHNOLOGY TO MANUFACTURE PERSONALIZED BONE CEMENT PLACEHOLDER MOLD FOR BONE DEFECT REPAIR AND RECONSTRUCTION WITH INFECTION: A CASE REPORT
2730
ENDOSCOPIC RESECTION AND DECOMPRESSION FOR LATERAL DISPLACEMENT OF CAGE AFTER OBLIQUE LUMBAR INTERBODY FUSION: A CASE REPORT FROM A SINGLE CENTER
2736
METALLOSIS-INDUCED CONVERSION SHOULDER ARTHROPLASTY: A UNIQUE EXPERIENCE AND LITERATURE REVIEW

WILEY

CHINESE ORTHOPAEDIC ASSOCIATION
中华医学会骨科学分会

View this journal online at wileyonlinelibrary.com/journal/os

### Orthopaedic Surgery

**Editor-in-Chief**
Gui-xing Qiu, *Beijing, China*
Francis John Hornicek, *Boston, USA*

**Associate Editor**
Yong-cheng Hu, *Tianjin, China*
Thomas Blattert, *Leipzig, Germany*
Douglas Letson, *Tampa, USA*
Xin-long Ma, *Tianjin, China*
Ling Qin, *Hong Kong, China*

**Editorial Board**
Giovanni Alessi, *Gent, Belgium*
Haluk Berk, *Izmir, Turkey*
Thomas Blattert, *Leipzig, Germany*
Mathis Bostrom, *New York, USA*
Kao-wha Chang, *Taiwan, China*
Edmund YS Chao, *Baltimore, USA*
Liao-bin Chen, *Wuhan, China*
Yan-xi Chen, *Shanghai, China*
Kenneth MC Cheung, *Hong Kong, China*
Thomas L. Clemens, *Birmingham, USA*
Benny Thellefsen Dahl, *Copenhagen, Denmark*
Lian-fu Deng, *Shanghai, China*
Jean Destandau, *Bordeaux, France*
Jian Dong, *Shanghai, China*
Shun-wu Fan, *Hangzhou, China*
David Fang, *Hong Kong, China*
Shi-jun Gao, *Shijiazhuang, China*
Wei Guo, *Beijing, China*
Yong Hai, *Beijing, China*
Alan R. Hargens, *San Diego, USA*
Francis John Hornicek, *Boston, USA*
William C. Horton, *Atlanta, USA*
John Hsiang, *Seattle, USA*
Yong-cheng Hu, *Tianjin, China*
Qing Jiang, *Nanjing, China*
Paul Jutte, *Groningen, Netherlands*
Jean Pierre Kalala, *Gent, Belgium*
Choon-Ki Lee, *Seoul, Korea*
Jean Jacque Lehuec, *Bordeaux, France*
Lawrence G. Lenke, *Seattle, USA*
Douglas Letson, *Tampa, USA*
Kwok Sui Leung, *Hong Kong, China*
Jian-hao Lin, *Beijing, China*
Bao-ge Liu, *Beijing, China*
Wei-hee Lo, *Taiwan, China*
William Weijia Lu, *Hong Kong, China*
Keith DK Luk, *Hong Kong, China*
Zong-ping Luo, *Suzhou, China*
Cyril Mauffrey, *Denver, USA*
Wei-hu Ma, *Ningbo, China*
Xin-long Ma, *Tianjin, China*
Ling Qin, *Hong Kong, China*
Gui-xing Qiu, *Beijing, China*
Axel Reinhardt, *Potsdam, Germany*
Pu-yi Sheng, *Guangzhou, China*
Harry L Shufflebarger, *Miami, USA*
David Stulberg, *Chicago, USA*
Paul Sung, *San Diego, USA*
Paul Unwin, *Leicester, UK*
Luc F. De Waele, *Gent, Belgium*
Fei Wang, *Shijiazhuang, China*
Wei-jun Wang, *Nanjing, China*
James N. Weinstein, *Chicago, USA*
Hee-Kit Wong, *Singapore*
Su-jia Wu, *Nanjing, China*
Jian-ru Xiao, *Shanghai, China*
Hui-lin Yang, *Suzhou, China*
Tzai-chiu Yu, *Taiwan, China*
Xi-jie Yu, *Chengdu, China*
Hansen A. Yuan, *New York, USA*
Hong-wen Zhu, *New York, USA*

**Address for Editorial Correspondence:** Editorial Board of *Orthopaedic Surgery*, Tianjin Hospital, 122 Munan Road, Tianjin 300050, China (email: orthopaedicsurgery@126.com; telephone: +86 22 2833 4734; fax: +86 22 2824 1184).

**Disclaimer:** The Publisher, Tianjin Hospital and Editors cannot be held responsible for errors or any consequences arising from the use of information contained in this journal; the views and opinions expressed do not necessarily reflect those of the Publisher, Tianjin Hospital and Editors, neither does the publication of advertisements constitute any endorsement by the Publisher, Tianjin Hospital and Editors of the products advertised.

**Copyright © 2023 The Authors. Orthopaedic Surgery published by Tianjin Hospital and John Wiley & Sons Australia, Ltd**

**Copyright and Copying (in any format)**
Copyright © 2023 The Authors. Orthopaedic Surgery published by Tianjin Hospital and John Wiley & Sons Australia, Ltd. All rights reserved. No part of this publication may be reproduced, stored or transmitted in any form or by any means without the prior permission in writing from the copyright holder. Authorization to copy items for internal and personal use is granted by the copyright holder for libraries and other users registered with their local Reproduction Rights Organisation (RRO), e.g. Copyright Clearance Center (CCC), 222 Rosewood Drive, Danvers, MA 01923, USA (www.copyright.com), provided the appropriate fee is paid directly to the RRO. This consent does not extend to other kinds of copying such as copying for general distribution, for advertising or promotional purposes, for republication, for creating new collective works or for resale. Permissions for such reuse can be obtained using the RightsLink “Request Permissions” link on Wiley Online Library. Special requests should be addressed to: permissions@wiley.com

For submission instructions, subscription and all other information visit: http://wileyonlinelibrary.com/journal/os

ISSN 1757-7861 (Online)

OS.JEB.October23
